# Previous dengue or Zika virus exposure can drive to infection enhancement or neutralisation of other flaviviruses

**DOI:** 10.1590/0074-02760190098

**Published:** 2019-08-12

**Authors:** Renato AS Oliveira, Edmilson F de Oliveira-Filho, Ana IV Fernandes, Carlos AA Brito, Ernesto TA Marques, Marli C Tenório, Laura HGV Gil

**Affiliations:** 1 Fundação Oswaldo Cruz, Instituto Aggeu Magalhães, Departamento de Virologia, Recife, PE, Brasil; 2 Universidade Federal da Paraíba, Departamento de Fisiologia e Patologia, João Pessoa, PB, Brasil; 3 Charité-Universitätsmedizin Berlin, Berlin, Germany; 4 Universidade Federal da Paraíba, Hospital Universitário Lauro Wanderley, Serviço de Doenças Infecciosas e Parasitárias, João Pessoa, PB, Brasil; 5 Universidade Federal da Paraíba, Escola Técnica de Saúde, Grupo de Estudos e Pesquisas em Imunologia Humana, João Pessoa, PB, Brasil; 6 Universidade Federal de Pernambuco, Departamento de Medicina Clínica, Recife, PE, Brasil; 7 University of Pittsburgh, Center for Vaccine Research, Department of Infectious Diseases and Microbiology, Pittsburgh, PA, USA

**Keywords:** ADE, flavivirus, dengue, Zika

## Abstract

**BACKGROUND:**

Dengue virus (DENV) has circulated in Brazil for over 30 years. During this time, one serotype has cyclically replaced the other, until recently, when all four distinct serotypes began to circulate together. Persistent circulation of DENV for long time periods makes sequential infections throughout a person’s life possible. After primary DENV infection, life-long immunity is developed for the infecting serotype. Since DENV and Zika virus (ZIKV) are antigenically similar, the possibility of cross-reactions has attracted attention and has been demonstrated *in vitro*.

**OBJECTIVE:**

The aim of this study was to investigate whether immune-sera from DENV and ZIKV infected patients would cross-react *in vitro* with other *Flaviviridae* family members.

**METHODS:**

Cross-reaction of the studied samples with yellow fever virus (YFV), West Nile virus (WNV), Rocio virus (ROCV), Saint Louis virus (SLEV) and Ilheus virus (ILHV) has been investigated by plaque reduction neutralisation test (PRNT) and the antibody-dependent enhancement (ADE) by flow-cytometry.

**FINDINGS:**

Antibodies against ZIKV and DENV virus cross-reacted with other flaviviruses either neutralising or enhancing the infection. Thus, viral entrance into FcRFcɣRII-expressing cells were influenced by the cross-reactive antibodies. ZIKV or DENV immune sera enhanced cellular infection by WNV, ILHV, ROCV and SLEV. Finally, DENV immune sera presented higher neutralising activity for YFV and SLEV. While ZIKV immune sera neutralised WNV, ILHV and ROCV with high frequencies of positivity.

**MAIN CONCLUSIONS:**

The co-circulation of those viruses in the same area represents a risk for the development of severe infections if they spread throughout the country. Successive flavivirus infections may have an impact on disease pathogenesis, as well as on the development of safe vaccine strategies.

Diseases caused by mosquito-borne flaviviruses, such as dengue virus (DENV), West Nile virus (WNV), yellow fever virus (YFV) and more recently Zika virus (ZIKV), are responsible for thousands of deaths and millions of hospitalisations worldwide each year.^1^ In 2015, ZIKV, associated with congenital Zika syndrome, emerged in Brazil. The outbreak introduced a new “player” into the complex epidemiologic Brazilian scenario where other members of the *Flaviviridae* family, such as dengue 1-4, were already co-circulating. Therefore, one of the biggest ongoing concerns remains therefore, whether cross-reactivity due to previous flavivirus infection or vaccination can protect from or intensify disease in ZIKV-infected patients.

Sera from DENV exposed human and animals have been demonstrated to cross-react and enhance ZIKV infection *in vitro*;^2^ ^,^ ^3^ ^,^ ^4^ ^,^ ^5^ nevertheless such cross-reactivity could neither be confirmed experimentally *in vivo* ^6^ nor in patients with acute ZIKV and a history of previous exposure to DENV.^7^

The aim of this study was to investigate whether immune-sera from DENV and ZIKV infected patients would cross-react *in vitro* with other *Flaviviridae* family members such as YFV, WNV, Rocio virus (ROCV), Ilheus virus (ILHV) and Saint Louis virus (SLEV), assessing how such cross-reactivity hinders or enhances infection.

## METHODS

*Cell and viruses -* BHK-21 and K562 (ATCC) cell lines were maintained respectively in minimum essential medium (MEM) and RPMI medium supplemented with 10% foetal bovine serum (FBS), 1% penicillin and 1% streptomycin and incubated at 37ºC with 5% CO_2_.

In the plaque reduction neutralisation test (PRNT) and antibody-dependent enhancement (ADE) assays the following strains were used: ZIKV PE243 strain, DENV-1, -2 and -4 (isolated from infected patients in the state of Pernambuco),^8^ DENV-3 (derived from an infectious clone),^9^ YFV 17D strain, YFV-prM/E-WNV, YFV-prM/E-ILHV, YFV-prM/E-SLEV and YFV-PrM/E-ROCV (viral chimeras built on a YFV 17D strain backbone where prM and E genes of YFV were exchanged for those of each flavivirus) (Gil et al., unpublished observation).

*Human serum samples -* A total of 19 sera were obtained from two cohort studies performed at the Universidade Federal da Paraíba (UFPB) (four samples) and at the Departamento de Virologia Instituto Aggeu Magalhães (IAM), Fundação Oswaldo Cruz (15 samples). Dengue and Zika fever cases were confirmed via virus isolation and/or viral RNA detection by reverse transcriptase-polymerase chain reaction (RT-PCR) for dengue and ZIKV as previously described^10^ ^-^ ^11^ and/or via positive serology for anti-DENV or anti-ZIKV IgM ELISA.^11^ Seroconversion was determined by the presence of circulating specific IgG antibodies. Primary or secondary infections were determined according to the presence/absence of circulating anti-dengue IgG at the beginning of the ZIKV infection (Table I). Primary anti-DENV positive samples were collected before the 2015 ZIKV outbreak. Samples were named as follows: (i) DENV - samples were IgG positive for DENV and IgG negative for ZIKV; (ii) ZIKV - samples with active ZIKV infection and IgG negative for DENV; and (iii) ZIKV/DENV - samples with active ZIKV infection and IgG positive for DENV. Written informed consent was obtained from all subjects and the study was approved by the ethic committees of the UFPB (protocol #032/2009/CEP/HULW/UFPB) and IAM (CEP: 11/11).

*Enhancement of virus infection in vitro assay -* An ADE assay was performed with DENV and ZIKV immune sera collected from patients to test the augmentation of infection in FcɣRII-expressing K562 cell lines. Briefly, sera were serially diluted (1:10 to 1:10240), added to virus aliquots with 8 × 10^4^ plaque forming units (PFU) for each studied virus and incubated for 20 minutes at room temperature. 1 × 10^5^ K562 cells were added per well of a 96 well plates in order to obtain an multiplicity of infection (MOI) of 0.8 and were incubated for 1 hour at 37ºC. Cells were centrifuged for 5 minutes at 450 *g* and 25ºC, after which supernatants were aspirated, fresh medium was added to the wells and plates were incubated for 48 hours at 37ºC, 5% CO_2_. Cells were stained using a mouse monoclonal antibody anti flavivirus E protein (hybridoma D1-4G2-4-15, ATCC HB-112), prepared as tissue culture supernatant from hybridomas obtained from ATCC. The immune complex was detected with an anti-mouse Ab conjugated to FITC (Sigma, Saint Louis, MO, USA). Fluorescent cells were then quantified by flow cytometry and results were expressed as percentage of positive events. ADE assay was performed in duplicate and repeated once.

*PRNT -* PRNT 90 % (PRNT_90_) assays were performed in BHK-21 cells, as previously described.^12^ Serial dilutions of previously positive DENV, ZIKV and DENV/ZIKV serum (1:10 to 1:5120) were mixed with 100 PFU of selected flaviviruses (ZIKV-PE243, YFV-17D, YFV-prM/E-WNV, YFV-prM/E-ILHV, YFV-prM/E-SLEV and YFV-PrM/E-ROCV), incubated for 1 hour at 37ºC, then added to the cells and incubated at 37ºC for an additional hour. After 1 hour of incubation, MEM containing 1.5 % carboxymethyl cellulose (CMC), 5% FBS and 1% penicillin-streptomycin were added to each well and plates were incubated at 37ºC, 5 % CO_2_ for 96 hours. After this incubation, plates were fixed with 10 % formaldehyde for 1 hour and subsequently stained with 1 % crystal violet for 15 minutes. Plaques were counted and the neutralisation capacity was estimated as the sera concentration causing a 90 % reduction in PFU. This assay was performed in duplicate and repeated once.

*Statistical analysis -* A nonparametric unpaired Mann-Whitney *U* test was used to compare the enhancing activity between tests and control. Results with p ≤ 0.05 were considered as statistically significant.

## RESULTS

*In vitro flavivirus neutralisation by convalescent-phase DENV, ZIKV and DENV/ZIKV immune sera -* Four DENV samples (P322, P122, P156, P234) presented high cross neutralisation titres for ZIKV (1:320, 1:320, 1:5120, 1:5120, respectively) (Table II). Besides, DENV positive sera also neutralised YFV [P132 (1:151), P491(1:261), P306 (1:1006)], WNV [P491 (1:252)], ILHV [P132 (1:320), P234 (1:1280)], SLEV [P156 (1:1007), P234 (1:1280)]. On the other hand, ZIKV samples neutralised ILHV [P05, P15, P67, P71 (1:20 for each)], YFV [P05, P67 (1:20 for each)], WNV [P05 (1:138), P13 (1:20), P67 (1:49), P69 (1:20)], SLEV [P71 (1:20)], ROCV [P15, P67 (1:20 each)] (Table II).

DENV positive sera presented neutralising activity for YFV and SLEV than ZIKV samples, the frequencies of positivity for YFV and SLEV were 55.6 and 33.4 percent, respectively (Table III). While the ZIKV samples neutralised WNV, ILHV and ROCV with frequencies of positivity of 40, 40 and 20 %, respectively (Table III).

**TABLE I:** Summary of dengue virus (DENV)- and Zica virus (ZIKV)-infected patients enrolled in the study

Patients ID	Age	Gender	ZIKV IgG	DENV IgG
P01	20	F	POS	NEG
P05	20	F	POS	NEG
P11	18	F	POS	NEG
P13	15	F	POS	NEG
P41	20	F	POS	NEG
P03	28	F	POS	POS (DV3,4)
P15	18	F	POS	POS (DV3,4)
P67	28	F	POS	POS (DV1,2,3,4)
P69	17	F	POS	POS (DV3,4)
P71	20	F	POS	POS (DV3,4)
P02	47	F	NEG	POS (DV2)
P132	37	M	NEG	NEG
P322	66	F	NEG	POS (DV1)
P481	16	M	NEG	POS (DV2)
P491	30	M	NEG	POS (DV3)
P306	47	F	NEG	POS (DV3)
P122	36	F	NEG	POS (DV4)
P156	64	M	NEG	POS (DV4)
P234	24	F	NEG	POS (DV4)

F: female; IgG: immunoglobulin G; M: male; NEG: negative; POS: positive.

**TABLE II:** Summary of plaque reduction neutralisation test (PRNT) titres and selected flavivirus infection enhancement by convalescent dengue virus (DENV)- and Zica virus (ZIKV)-infected samples

	Sample ID	ZIKV		DENV1	DENV2	DENV3	DENV4	YFV		WNV		ILHV		SLEV		ROCV	
ZIKV IgG+		PRNT		PRNT	PRNT	PRNT	PRNT	PRNT	ADE	PRNT	ADE	PRNT	ADE	PRNT	ADE	PRNT	ADE
	P01	2560		<20	<20	<20	<20	<20	1.61	<20	2.00	<20	2.33	<20	2.61	<20	3.20
	P05	12980		<20	<20	<20	<20	20	1.12	138	1.33	20	1.81	<20	2.26	<20	1.47
	P11	634		<20	<20	<20	<20	<20	1.19	<20	1.60	<20	1.59	<20	1.50	<20	1.69
	P13	1575		<20	<20	<20	<20	<20	0.67	20	1.47	<20	3.03	<20	2.78	<20	0.81
	P41	343		<20	<20	<20	<20	<20	1.32	<20	1.82	<20	3.95	<20	4.19	<20	3.53
	P03	2560		<20	<20	320	80	<20	1.21	<20	2.36	<20	2.90	<20	3.64	<20	4.65
	P15	1280		<20	<20	80	320	<20	1.38	<20	2.36	20	3.82	<20	3.98	20	3.84
	P67	3729		63	67	340	2192	20	1.09	49	0.95	20	1.07	<20	1.44	20	2.25
	P69	536		<20	<20	570	117	<20	0.76	20	1.03	<20	2.42	<20	2.14	<20	1.90
	P71	232		<20	<20	76	215	<20	0.97	<20	0.73	20	2.97	20	2.77	<20	2.58
ZIKV/DENV IgG-	P132	<20	1.75	<20	<20	<20	<20	151	1.21	20	1.54	320	1.45	<20	1.79	<20	3.89
DENV IgG+	P02	<20	3.31	<20	5120	<20	<20	<20	1.30	<20	1.60	<20	1.50	<20	2.10	<20	0.90
	P322	320	0.75	5120	<20	<20	<20	20	1.32	<20	2.84	<20	0.91	<20	1.08	<20	2.02
	P481	<20	5.38	1280	5120	1280	<20	<20	1.73	<20	1.90	<20	5.21	20	4.82	<20	4.06
	P491	<20	3.00	320	40	>2560	<20	261	1.30	252	2.57	<20	2.03	<20	2.38	<20	2.59
	P306	<20	1.75	640	160	>2560	<20	1006	1.12	<20	1.63	<20	2.02	<20	3.09	<20	4.90
	P122	320	1.00	280	5120	80	5120	20	1.20	<20	1.17	<20	1.21	<20	1.80	<20	1.71
	P156	5120	0.38	320	320	80	1280	<20	1.42	<20	1.92	<20	1.16	1007	1.28	<20	0.06
	P234	5120	0.38	<20	1280	1280	5120	<20	1.08	<20	0.87	1280	0.73	1280	1.91	<20	1.11

ADE: antibody dependent enhancement; IgG: immunoglobulin G; ILHV: Ilheus virus; ROCV: Rocio virus; SLEV: Saint Louis encephalitis virus; WNV: West Nile virus; YFV: yellow fever virus.

**TABLE III:** Frequency of positivity

	PRNT_90_ (%)										
		ZIKV	DENV1	DENV2	DENV3	DENV4	YFV	WNV	ILHV	SLEV	ROCV
ZIKV IgG+	Positives	10	1	1	5	5	2	4	4	1	2
	(n)	10	10	10	10	10	10	10	10	10	10
	(%)	100	10	10	50	50	20	40	40	10	20
DENV IgG +	Positives	4	6	7	6	3	5	2	2	3	0
	(n)	9	9	9	9	9	9	9	9	9	9
	(%)	44.4	66.7	77.8	66.7	33.4	55.6	22.2	22.2	33.4	0

DENV: dengue virus; IgG: immunoglobulin G; ILHV: Ilheus virus; PRNT: plaque reduction neutralisation test; ROCV: Rocio virus; SLEV: Saint Louis encephalitis virus; WNV: West Nile virus; YFV: yellow fever virus; ZIKV: Zika virus.

*DENV or ZIKV immune sera deliver flavivirus virions to FcRFcɣRII-expressing cells -* We tested the ability of DENV or ZIKV convalescent sera to promote ADE in the FcRFcɣRII-expressing cell line K562. ZIKV was preincubated with titrered convalescent anti-dengue serum and selected flaviviruses (ZIKV-PE243, YFV-17D, YFV-prM/E-WNV, YFV-prM/E-ILHV, YFV-prM/E-SLEV and YFV-prM/E-ROCV) were preincubated with convalescent DENV, ZIKV and ZIKV/DENV sera and then used to infect K562 cells. In all but three cases DENV sera increased ZIKV infection with a median 1.75-fold increase of infection by ZIKV-PE243 (Fig. A).

Six representative samples were serially diluted in order to assess whether the presence of neutralising antibodies against DENV samples would interfere with ZIKV entrance into K562 cells. Three samples at 1:10 dilution were shown to block (P122, P156 and P322) and three samples were shown to enhance (P02, P481 and P491) ZIKV entrance. With the increase of the dilution factor, neutralising samples became infection enhancers while the other samples reduced the number of positive cells (Fig. B). Thus, cross-reactive anti-DENV antibodies lead either to ADE or to neutralisation.

In general, DENV, ZIKV and ZIKV/DENV positive sera samples cross-reacted with WNV, YFV, SLEV, ROCV and ILHV, enhancing viral entrance in K562 cells (Fig. C-G). Specially, this held true for SLEV, ROCV and ILHV, which presented the highest levels of entrance (Fig. E-G). YFV infection was slightly increased in five samples (Fig. D), while WNV infection was increased in association with DENV and ZIKV samples (Fig. C).

**Figure:**
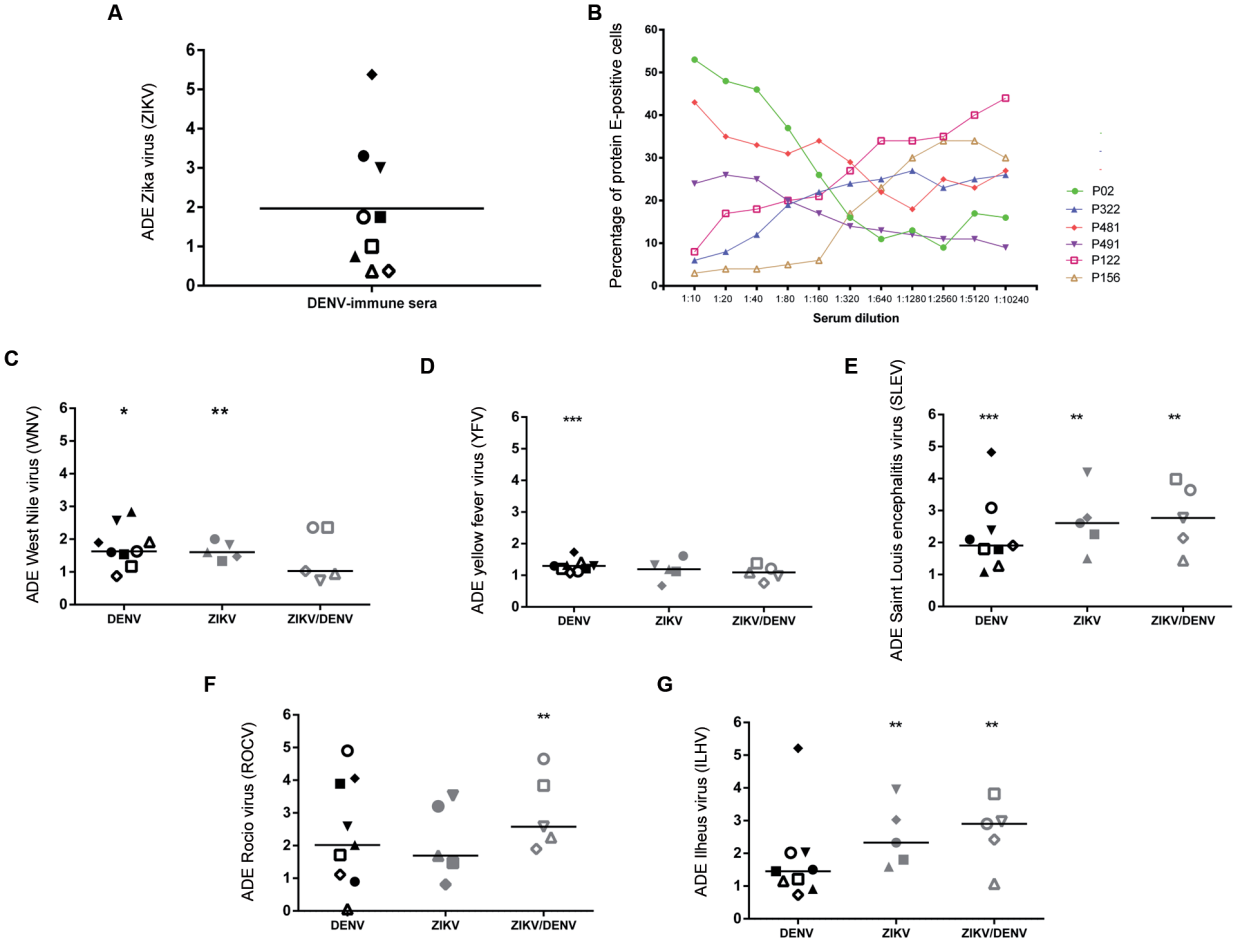
Flavivirus enhancement of infection by DENV- and ZIKV-immune sera. (A) enhancement of DENV-immune sera (n = 9) on ZIKV infection in K562 cells. horizontal bars indicate the median values; (B) six representative antibody dependent enhancement (ADE) curves (percentage of positive cells) of K562 cells infected with ZIKV-PE243; Infection enhancement of (C) YFV-prM/E-WNV; (D) YFV-17D; (E) YFV-prM/E-SLEV; (F) YFV-prM/E-ROCV; and (G) YFV-prM/E-ILHV chimeras by convalescent sera from positive DENV (n = 09), ZIKV (n = 5) and ZIKV/DENV patients (n = 5) donors was evaluated. Double comparisons with control group were performed by Mann Withney U test. *: p ≤ 0.05; **: p ≤ 0.01; ***: p ≤ 0.001.

## DISCUSSION

Immunological cross-reactivity amongst members of the *Flaviviridae* family has been well described. Flaviviruses are immunologically and genetically related, with high levels of conservation of structural proteins E and prM, the main targets for adaptive immune responses.^13^ ^,^ ^14^ ADE was initially described in secondary DENV infections, but may also occur among other flavivirus infections, influencing the disease pathogenesis, as well as the design of new vaccines against those flaviviruses.

Despite *in vivo* studies have demonstrated that previous DENV exposure does not result in more severe ZIKV infection,^6^ ^,^ ^7^ we observed that controlled ZIKV infection of K562 cells was enhanced by DENV immune sera and our findings are in agreement with other previous reports.^2^ ^,^ ^3^ The discrepancies between the different *in vivo* and *in vitro* assays might be owed to the rare occurrence of ADE,^15^ once only few patients progress to severe dengue disease. In addition, the conditions outlined in *in vitro* experimentation are more limited with respect to cell interaction. Under *in vivo* conditions, a very large variety of cells can interact with the ZIKV and antibodies in the same environment and how this interaction may influence viral infectivity is still unclear. ADE should thus not be neglected and, despite the number of studies already available, it should be extensively explored, especially given the landscape of ongoing DENV vaccine trials.

Cross-reactivity is a hallmark of flavivirus infection due to antigenic similarity of these viruses, whether protection or infection enhancement will be displayed depends on the quantity and quality of pre-existing cross-reactive antibodies.^16^ DENV serotypes present similarities in the envelope (E) protein at the amino acid sequence level; however they are antigenically different, due to amino acid differences that lead to structural changes in protein E viral protein.^17^ In our results, we observed that sera from patients infected with DENV 1 and 4 serotypes neutralised ZIKV cell entrance, while sera from patients infected with DENV 2 and 3 serotypes enhanced ZIKV cell entrance. One possible reason for that could be the antigenic similarity/difference between DENV different serotypes and ZIKV. In fact, other study demonstrated that anti-DENV 3 immune sera cross-reacted more with ZIKV than another serotypes.^18^

The neutralising ability of immune sera is directly influenced by the maturation stage of the viral particle, as observed in a previous study.^19^ This phenomenon may have occurred in our study, both in the neutralisation studies of isolates and of the chimera and infectious clone. We can make this inference, since the infectious clones used in this study were previously characterised phenotypically and genetically, presenting similar replicative behaviour to the virus isolated.

In order to expand existing cross-reactivity studies, we tested the ability of sera from Dengue- and Zika-convalescent patients to recognise selected flaviviruses. Antibodies elicited against ZIKV enhanced or neutralised the flavivirus infections in different proportions. In this context, it is important to highlight that previous DENV infection did not interfere significantly with ZIKV enhancement.

YFV cellular entrance was slightly increased by ZIKV and DENV samples, especially by DENV patient’s sera. Therefore, circulating antibodies against DENV or ZIKV may not present an important threat for future YFV infection. This observation is especially important in places with known past or ongoing YFV outbreaks, as recently observed in Brazil.^20^ Is important to state that the samples employed in this study were collected in Northeast Region of Brazil, where high levels of microcephaly cases associated with ZIKV recent outbreak were reported. In addition, most of the local population is not protected for YFV, since vaccination is not mandatory in this region. On the other hand, lack of YFV protection could be associated with congenital malformations observed in the last ZIKV outbreak in this region. Since, recent studies observed that vaccination against YFV led to the prevention of neurological problems in ZIKV infected mice.^21^

Bardina et al.^2^ demonstrated that the presence of anti-WNV antibodies increased levels of ZIKV infection in cell lines and in mice, the latter being evidenced by the increase of viral particles in animal tissues; they suggested that previous WNV infection would make ZIKV infection more severe. We however, demonstrate that the presence of anti-ZIKV antibodies increases WNV infection.

Cross-reaction may not be harmful to patients and certain samples also induced cross-protection with neutralisation titres ranging from 20 to 1,280 in a PRNT_90_. Neutralisation may lead to nonspecific infection control producing asymptomatic infections. As observed by Amarilla et al.^22^ When reported that previous exposure to Ilheus and Saint Louis encephalitis viruses elicited cross-protection against ROCV infection in mice.

In the present study, we demonstrated that antibodies generated against ZIKV or DENV neutralised or enhanced the entrance of ROCV, SLEV, WNV and ILHV in K562 cells. Serological evidences showed those flaviviruses to be circulating in Brazil^22^ ^,^ ^23^ and, considering that DENV and ROCV are endemic, the co-circulation of those viruses represents a risk for the development of severe infections if they spread throughout the country. Extensive *in vivo* studies should be undertaken to validate the actual potential of successive flavivirus infections for becoming increasingly severe as a result of cross-immunological reactions.

## References

[1] Mayer SV, Tesh RB, Vasilakis N (2017). The emergence of arthropod-borne viral diseases a global
prospective on dengue, chikungunya and zika fevers. Acta Trop.

[2] Bardina SV, Bunduc P, Tripathi S, Duehr J, Frere JJ, Brown JA (2017). Enhancement of Zika virus pathogenesis by preexisting
antiflavivirus immunity. Sci.

[3] Castanha PMS, Nascimento EJM, Braga C, Cordeiro MT, de Carvalho OV, de Mendonça LR (2017). Dengue virus-specific antibodies enhance Brazilian Zika virus
infection. J Infect Dis.

[4] Dejnirattisai W, Supasa P, Wongwiwat W, Rouvinski A, Barba-Spaeth G, Duangchinda T (2016). Dengue virus sero-cross-reactivity drives antibody-dependent
enhancement of infection with Zika virus. Nat Immunol.

[5] Priyamvada L, Quicke KM, Hudson WH, Onlamoon N, Sewatanon J, Edupuganti S (2016). Human antibody responses after dengue virus infection are highly
cross-reactive to Zika virus. Proc Natl Acad Sci United States Am.

[6] Pantoja P, Pérez-Guzmán EX, Rodríguez IV, White LJ, González O, Serrano C (2017). Zika virus pathogenesis in rhesus macaques is unaffected by
pre-existing immunity to dengue virus. Nat Commun.

[7] Terzian ACB, Schanoski AS, Mota MT de O.da Silva RA.Estofolete CF.Colombo TE (2017). Viral load and cytokine response profile does not support
antibody-dependent enhancement in dengue-primed Zika virus-infected
patients. Clin Infect Dis.

[8] Cordeiro MT, Schatzmayr HG, Nogueira RMR, Oliveira de VF, Melo de WT, Carvalho de EF (2007). Dengue and dengue hemorrhagic fever in the State of Pernambuco,
1995-2006. Rev da Soc Bras de Med Trop.

[9] Santos JJS, Cordeiro MT, Bertani GR, Marques ETA, Gil LHVG (2014). A two-plasmid strategy for engineering a dengue virus type 3
infectious clone from primary Brazilian isolate. An Acad Bras Ciencias.

[10] Lanciotti RS, Calisher CH, Gubler DJ, Chang GJ, Vorndam AV (1992). Rapid detection and typing of dengue viruses from clinical
samples by using reverse transcriptase-polymerase chain
reaction. J Clin Microbiol.

[11] Cordeiro MT, Brito CAA, Pena LJ, Castanha PMS, Gil LHVG, Lopes KGS (2016). Results of a Zika virus (ZIKV) immunoglobulin M-specific
diagnostic assay are highly correlated with detection of neutralizing
anti-ZIKV antibodies in neonates with congenital disease. J Infect Dis.

[12] Roehrig JT, Hombach J, Barrett AD (2008). Guidelines for plaque-reduction neutralization testing of human
antibodies to dengue viruses. Viral Immunol.

[13] Priyamvada L, Hudson W, Ahmed R, Wrammert J (2017). Humoral cross-reactivity between Zika and dengue viruses
implications for protection and pathology. Emerg Microbes & Infect.

[14] Reynolds CJ, Suleyman OM, Ortega-Prieto AM, Skelton JK, Bonnesoeur P, Blohm A (2018). T cell immunity to Zika virus targets immunodominant epitopes
that show cross-reactivity with other flaviviruses. Sci Reports.

[15] George J, Valiant WG, Mattapallil MJ, Walker M, Huang Y-JS, Vanlandingham DL (2017). Prior exposure to Zika virus significantly enhances peak dengue-2
viremia in Rhesus macaques. Sci Reports.

[16] Katzelnick LC, Montoya M, Gresh L, Balmaseda A, Harris E (2016). Neutralizing antibody titers against dengue virus correlate with
protection from symptomatic infection in a longitudinal
cohort. Proc Natl Acad Sci United States Am.

[17] Katzelnick LC, Fonville JM, Gromowski GD, Bustos Arriaga J, Green A, James SL (2015). Dengue viruses cluster antigenically but not as discrete
serotypes. Sci.

[18] Andrade P, Gimblet-Ochieng C, Modirian F, Collins M, Cárdenas M, Katzelnick LC (2019). Impact of pre-existing dengue immunity on human antibody and
memory B cell responses to Zika. Nat Commun.

[19] Dowd KA, Mukherjee S, Kuhn RJ, Pierson TC (2014). Combined effects of the structural heterogeneity and dynamics of
flaviviruses on antibody recognition. J Virol.

[20] Goldani LZ (2017). Yellow fever outbreak in Brazil, 2017. Braz J Infect Dis.

[21] Kum DB, Mishra N, Boudewijns R, Gladwyn-Ng I, Alfano C, Ma J (2018). A yellow fever-Zika chimeric virus vaccine candidate protects
against Zika infection and congenital malformations in mice. NPJ VCaccines.

[22] Amarilla AA, Fumagalli MJ, Figueiredo ML, Lima-Junior DS, Santos-Junior NN, Alfonso HL (2018). Ilheus and Saint Louis encephalitis viruses elicit
cross-protection against a lethal Rocio virus challenge in
mice. PloS One.

[23] de Oliveira-Filho EF, Oliveira RAS, Ferreira DRA, Laroque PO, Pena LJ, Valença-Montenegro MM (2018). Seroprevalence of selected flaviviruses in free-living and
captive capuchin monkeys in the state of Pernambuco, Brazil. Transbound Emerg Dis.

